# Understanding How to Improve the Use of Clinical Coordination Mechanisms between Primary and Secondary Care Doctors: Clues from Catalonia

**DOI:** 10.3390/ijerph18063224

**Published:** 2021-03-20

**Authors:** Laura Esteve-Matalí, Ingrid Vargas, Franco Amigo, Pere Plaja, Francesc Cots, Erick F. Mayer, Joan-Manuel Pérez-Castejón, María-Luisa Vázquez

**Affiliations:** 1 Health Policy and Health Services Research Group, Health Policy Research Unit, Consortium for Health Care and Social Services of Catalonia, 08022 Barcelona, Spain; lesteve@consorci.org (L.E.-M.); famigo@imim.es (F.A.); mlvazquez@consorci.org (M.-L.V.); 2 Department for Paediatrics, Obstetrics and Gynaecology, Preventive Medicine, Universitat Autònoma de Barcelona, 08193 Bellaterra, Spain; 3 Health Services Research Unit, IMIM-Institut Hospital del Mar d’Investigacions Mèdiques, 08003 Barcelona, Spain; 4 Fundació Salut Empordà, 17600 Figueres, Spain; pplaja@salutemporda.cat; 5 Parc de Salut Mar, 08019 Barcelona, Spain; FCots@parcdesalutmar.cat; 6 Serveis de Salut Integrats Baix Empordà, 17230 Palamós, Spain; emayer@ssibe.cat; 7 Badalona Serveis Assistencials, 08911 Badalona, Spain; jpcastejon@bsa.cat

**Keywords:** clinical coordination, coordination mechanisms, primary care, secondary care, electronic medical record, remote consultation, health services research, questionnaire

## Abstract

Clinical coordination between primary (PC) and secondary care (SC) is a challenge for health systems, and clinical coordination mechanisms (CCM) play an important role in the interface between care levels. It is therefore essential to understand the elements that may hinder their use. This study aims to analyze the level of use of CCM, the difficulties and factors associated with their use, and suggestions for improving clinical coordination. A cross-sectional online survey-based study using the questionnaire COORDENA-CAT was conducted with 3308 PC and SC doctors in the Catalan national health system. Descriptive bivariate analysis and logistic regression models were used. Shared Electronic Medical Records were the most frequently used CCM, especially by PC doctors, and the one that presented most difficulties in use, mostly related to technical problems. Some factors positively associated with frequent use of various CCM were: working full-time in integrated areas, or with local hospitals. Interactional and organizational factors contributed to a greater extent among SC doctors. Suggestions for improving clinical coordination were similar between care levels and related mainly to the improvement of CCM. In an era where management tools are shifting towards technology-based CCM, this study can help to design strategies to improve their effectiveness.

## 1. Introduction

Clinical coordination across care levels is an inherent challenge for health systems, especially for those based on primary care such as the Spanish national health system. Its relevance is growing in step with the current context of increasing medical specialization and continuous advances in technology, together with the rise in chronic disease prevalence [1,2]. Cross-level clinical coordination mechanisms can play an important role in addressing this challenge [3,4] due to their potential to improve the transfer of clinical information and communication between primary care (PC) and secondary care (SC) doctors [5,6], thus reducing delays, duplication of exams and medical errors, as well as SC patient consultations and waiting times [7,8,9,10]. Despite their importance, previous studies highlight the limited use (or problems in the use) of cross-level coordination mechanisms, both at a national and international level [11,12,13,14], suggesting that they have not been adopted as expected. Health systems need to gain further understanding of the factors and difficulties influencing the utilization of coordination mechanisms in order to adapt them and improve their adoption rates and thus, their effectiveness.

Health services can implement two different types of cross-level coordination mechanisms according to the situation and the available resources: feedback mechanisms and standardization mechanisms [15]. Feedback mechanisms are based on communication and exchange of information between professionals and include mutual adjustment mechanisms such as the vertical information systems (e.g. shared Electronic Medical Records—EMR) and direct communication tools, which can be synchronous (phone consultations) or asynchronous (virtual consultations through EMR or email) [15]. These mechanisms are especially useful in situations where there is a high volume of information to be processed, and for interdependent and highly specialized activities [16]. Standardization mechanisms are based on programming and aim to coordinate by systematizing processes (such as shared protocols or clinical guidelines), outcomes (plans), or health professionals’ skills (such as clinical training). They are considered useful for situations that can be anticipated and do not require a rapid response [15,16]. Care coordination mechanisms like joint clinical case conferences combine feedback based on direct communication with standardization of skills.

Due to the rapid progress of information and communication technology, in recent decades there has been an increasing tendency to implement feedback mechanisms based on new technologies [17,18,19], with particular emphasis on asynchronous mechanisms [6,12]. However, the characteristics and successes of their rollout have varied widely across regions, services and professionals [6], with inconsistent implementation due to technological challenges or other unintended consequences [17,20].

The use of coordination mechanisms by health professionals can be influenced by many factors related to the characteristics of the context in which the mechanism is implemented, including organizational and individual factors, such as technical limitations, work overload, lack of time or mistrust [21,22]. However, evidence on the factors [23,24,25] and barriers [12,26,27] hindering the use of care coordination mechanisms generally focuses on a single type of mechanism, despite the fact that these factors and barriers may influence the use of each coordination mechanism differently [28], and that in real life conditions, health organizations use a combination of mechanisms, which may influence each other, and hence require a more comprehensive analytical approach. Moreover, although previous studies suggest that experiences and perceptions of clinical coordination differ between levels of care [14], studies analysing the use of coordination mechanisms normally concentrate on a single care level, either PC or SC, even though PC doctors are expected to make more use of coordination mechanisms than SC doctors, due to their greater need to communicate as patient care coordinators [29,30], and different factors may influence their use depending on the level of care. Besides, studies exploring suggestions for improving clinical coordination from the point of view of PC and SC doctors are inexistent to date, and would provide crucial information for the successful implementation of effective reforms to promote clinical coordination.

The national health system (SNS) of Catalonia, financed by taxes and with almost universal coverage and free access at points of delivery, is PC-based, which means that PC acts as the gatekeeper and coordinator of patient care and SC plays a consultancy role for PC and is responsible for more complex care. The SNS is geographically organized into healthcare areas containing the PC and SC resources (PC centres, acute and long-term hospitals) needed to attend to their population, and PC is in turn divided into basic health zones, which are the smallest units of health care. The provision of services is the responsibility of a variety of providers: a public company—the Catalan Health Institute, but also public consortia, and municipal and private foundations, mostly non-profit [31]. The organization of the SNS together with the diversity of providers has generated different PC/SC provider management models, and also great variability in the implementation of cross-level coordination mechanisms [13]. According to the management type, healthcare areas can be classified into: integrated, where a single entity manages SC and the majority of PC centres; semi-integrated, where one entity manages SC and some PC centres; and non-integrated, where different entities manage SC and PC centres [32]. Cross-level coordination mechanisms also differ in terms of the level responsible for their implementation: they might be implemented at a regional level, such as the shared EMR of Catalonia (HC3) or clinical guidelines, or developed by the managing entities at a local level, such as the shared EMR of the organization (HCC) or joint clinical case conferences [13].

Previous qualitative and quantitative studies conducted in the Catalan health system show limited use of cross-level coordination mechanisms and suggest that this could be related to organizational factors [13,14,33]. However, there are no comprehensive evaluations on the use of existing coordination mechanisms. To address this gap in knowledge, the objective of this study, which forms part of a wider study [14,34], is to analyze the level of use of cross-level clinical coordination mechanisms, the difficulties and factors associated with their use, and suggestions for improvement of clinical coordination in the Catalan health system.

## 2. Methods

### 2.1. Study Design and Study Areas

A cross-sectional study was conducted based on an online survey, utilizing the self-administered questionnaire COORDENA-CAT, with primary care (PC) and secondary care (SC) doctors in the national health system of Catalonia. The study areas were defined based on the basic health zones and the corresponding referral hospitals (acute and long-term care) which make up the healthcare areas.

### 2.2. Study Population and Sample

The study population consisted of PC and SC doctors in the national health system of Catalonia. Inclusion criteria were: having at least one year of experience in the organization, daily practice involving contact with doctors of the other care level and direct contact with patients. A total of 15,813 doctors from 41 study areas were invited to participate, and the final sample was of 3308 doctors from 32 areas (21% participation rate).

### 2.3. Data Collection and Questionnaire

Data were collected between October and December of 2017, through the self-administered COORDENA.CAT questionnaire, which had been previously adapted, piloted, and validated [34]. Invitations to participate were sent to all doctors meeting the inclusion criteria by their organization through the institutional e-mail system, containing a link giving anonymous access to the questionnaire. The questionnaire measures doctors’ experience of clinical coordination across care levels and the factors influencing it, their knowledge, use, and difficulties in use of cross-level coordination mechanisms, and suggestions for improving clinical coordination. These last two sections are the focus of this paper. Details on the adaptation, validation and structure of the questionnaire and on data collection have already been published [14,34].

### 2.4. Variables

The outcome variables were the frequent use (elicited by a closed question) of six cross-level clinical coordination mechanisms (shared Electronic Medical Record (EMR) of Catalonia (HC3), shared EMR of the organization (HCC), virtual consultation through EMR, email or phone and joint clinical case conferences) and the existence and types of difficulties in their use (elicited by a multiple choice question with an open-ended option), as well as suggestions for improving cross-level clinical coordination (elicited by an open-ended question). Frequent use was defined as “daily” for the shared EMRs, “daily or weekly” for the virtual consultation through EMR, email or phone, and “daily, weekly or monthly” for joint clinical case conferences. The existence of difficulties was dichotomized (no = ”no difficulty”/yes = ”any difficulty”). The level of care was a stratification variable.

The explanatory variables to explore factors associated with the frequent use of coordination mechanisms were selected based on the theoretical framework on care coordination and coordination mechanisms underlying this study [35], and were as follows: sociodemographic, employment characteristics, attitude towards work, interactional factors between doctors, type of area, and organizational factors (Table 1).

### 2.5. Analyses

Univariate and bivariate analyses were performed to describe the outcome and explanatory variables by level of care. Chi-square tests were performed to determine statistically significant differences between PC and SC doctors (*p*-value < 0.05 as statistically significant). A content analysis of the answers to the open-ended questions was conducted, whereby the different answers were identified and coded, frequencies were calculated, and similar codes were grouped into larger code groups and stratified by level of care. Finally, to explore the factors associated with the frequent use of each coordination mechanism, logistic regression models were carried out for each level of care. Robust covariance adjustments –employing type of area according to type of management– were used to account for correlated observations due to clustering. Raw and adjusted odds ratios (OR) at the 95% confidence interval (95% CI) were calculated. The final models were reached by introducing the variables by groups, keeping the statistically significant and theoretically relevant ones. Multicollinearity between variables was assessed by a correlation matrix and by the variance inflation factor (VIF), and the Hosmer-Lemeshow test was used to assess the fitness of the models. The statistical software used was STATA 15.

## 3. Results

### 3.1. Sample Description

Sample characteristics were similar for both levels of care, but with some differences. The majority of doctors in both care levels were women but in a higher proportion in PC (PC 68.99%; SC 52.06%), and were between 41 and 55 years old (PC 51.21%; SC 43.90%), born in Spain (87.37%) and trained in a clinical speciality, particularly in PC (PC 98.86%; SC 67.32%). In terms of employment characteristics, in both care levels, the highest proportion had 11 to 20 years’ work experience (PC 34.06%; SC 32.88%), a permanent contract (PC 96.31%; SC 88.10%) and worked full-time (91.88%). With regard to attitude and to the type of area, most doctors were satisfied with their job (PC 82.24%; SC 86.17%); a higher proportion (PC 41.81%; SC 45.22%) worked in an integrated area; and a higher proportion of PC (67.48%) than of SC doctors (50.16%) worked in areas with local or regional hospitals (Table 1).

In terms of interactional factors, most doctors in both levels (71.01%) reported having a positive previous experience of cross-level clinical coordination. Although less than half, and in lower proportion in SC (PC 44.02%; SC 36.32%), reported knowing the doctors of the other care level, the great majority, especially in PC (PC 97.20%; SC 83.70%), reported trusting in their clinical skills. The majority, higher in SC than PC (PC 66.19%; SC 85.56%), perceived that the care they provide influences the practice of doctors of the other level, while most doctors, more in PC than SC (PC 95.19%; SC 74.01%), considered that PC doctors are responsible for the coordination of the patient through the continuum of care (Table 1). With regard to organizational factors, there were fewer differences between levels. More than half of the doctors, but in a higher proportion in PC, found that their organization’s management facilitates coordination across levels (PC 67.44%; SC 53.88%) and that it sets objectives aimed at clinical coordination (55.20%). In contrast, a small proportion (13.90%) reported having enough time to dedicate to coordination during their working day, and 21.44% of SC doctors indicated that they also conduct patient consultations in a PC centre (Table 1).

### 3.2. Level of Use and Factors Associated with the Frequent Use of Clinical Coordination Mechanisms

The level of use reported by doctors that had access to the coordination mechanisms was different according to the mechanism and level of care (Table 2). Feedback mechanisms based on vertical information systems were the most frequently used, particularly the shared EMR of the organization (HCC, PC 94.04%; SC 74.29%), followed by the shared EMR of Catalonia (HC3, PC 77.34%; SC 59.65%), both in a higher proportion among PC doctors. Moreover, around half of the doctors reported frequent use of virtual consultations through EMR (52.36%) while a much smaller proportion, slightly higher among SC doctors, reported frequent consultations via email (PC 23.31%; SC 28.27%) and phone (PC 12.80%; SC 26.40%). Finally, more than half of the doctors reported frequent participation in joint clinical case conferences, but in a higher proportion among PC doctors (PC 69.87%; SC 54.46% SC) (Table 2).

The factors associated with frequent use of coordination mechanisms also varied according to the type of mechanism and level of care (Table 3, Table 4 and Table 5). With regard to the shared EMRs (HC3 and HCC) (Table 3), factors positively associated among SC doctors only were working in a clinical speciality, having less than 10 years of experience as a doctor and knowing the doctors of the other level personally; for HC3 only, having a full-time contract; and for HCC only, perceiving that their own practice influences the other level. Working in a high technology hospital was negatively associated among SC doctors for both EMRs, and perceiving that the organization’s management facilitates coordination was also negatively associated for HC3. On the other hand, among PC doctors and for both EMRs, having less than 10 years of experience was negatively associated; for HC3 only, negatively associated factors were also being satisfied with their job in the organization, a positive previous experience of coordination, the perception that their own practice influences the other level and having time to dedicate to coordination; and for HCC only, knowing the doctors of the other care level personally and the perception that the organization’s management facilitates coordination. Finally, both for PC and SC, working in an integrated area was negatively associated with HC3 but positively with HCC.

In terms of the different types of cross-level consultations (EMR, email and phone) (Table 4), factors positively associated with frequent use among SC doctors were being male, working in a clinical speciality, having less experience as a doctor, knowing the doctors of the other level personally, and perceiving that their organization sets objectives aimed at coordination; for consultations via EMR only, conducting consultations in a PC centre; and for phone consultations only, having a temporary contract and perceiving that their own practice influences the other level. Perceiving that the organization’s management facilitates coordination was negatively associated among SC doctors for email and phone consultations. In the case of PC doctors, one factor positively associated with frequent use of consultations via EMR was feeling responsible for the coordination of patient care and, in the case of email consultations, also having less experience as a doctor, trusting in the clinical skills of the doctors of the other level, and perceiving that their own practice influences the other level. Finally, factors negatively associated with all consultation types for both care levels were working in a high technology hospital and, for consultations via EMR among SC doctors and via phone among PC doctors, working in an integrated area.

Factors associated with frequent participation in joint clinical case conferences differed considerably between care levels (Table 5). Among SC doctors, negatively associated factors were being male and working in a high-technology hospital, whereas working full-time, knowing the doctors of the other care level and conducting consultations in a PC centre were positively associated. Among PC doctors, having a positive previous coordination experience was a positively associated factor whereas trusting in the clinical skills of the doctors of the other care level was negatively associated. For both care levels, working in a non-integrated area was positively associated.

### 3.3. Difficulties in Use of Cross-Level Clinical Coordination Mechanisms

**Figure 1:**
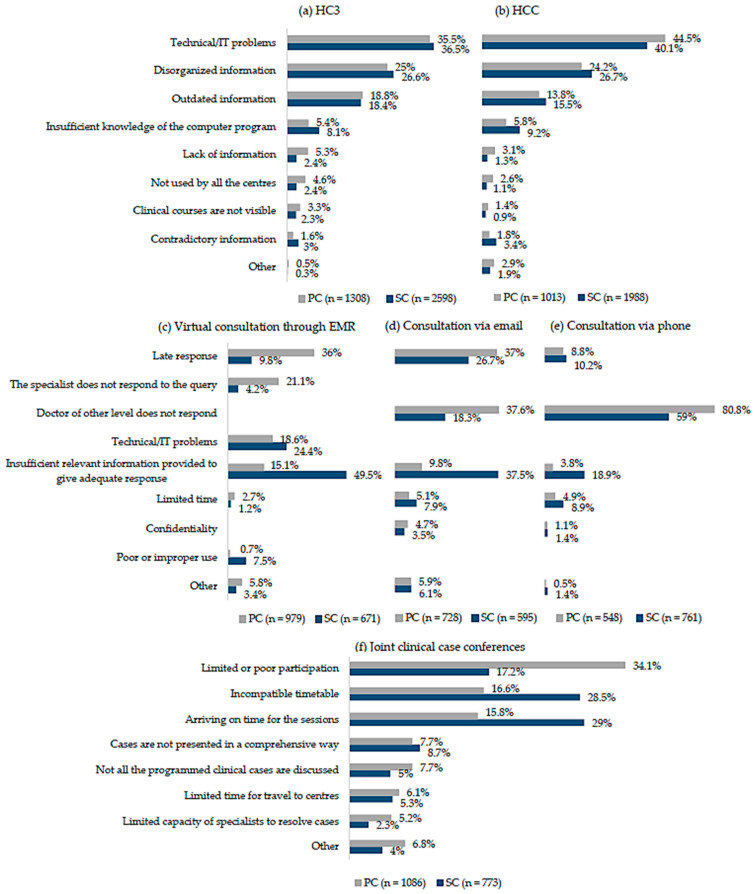
Difficulties in use of cross-level clinical coordination mechanisms, by level of care: (**a**) Shared EMR of Catalonia (HC3); (**b**) Shared EMR of the organization (HCC); (**c**) Virtual consultation through EMR; (**d**) Consultation via email; (**e**) Consultation via phone; (**f**) Joint clinical case conferences; *n*: total of answers.

More than half of the doctors of both care levels that used the coordination mechanisms reported difficulties, but with differences according to the mechanism (more for HC3 and joint clinical case conferences and fewer for consultation via phone) and level of care (Table 2, Figure 1). Most doctors reported difficulties when using the EMRs, but in a higher proportion for HC3 (76.89%) than for HCC (66.05%) (Table 2). The most frequent difficulties, over the totality of answers, encountered for both EMRs and both care levels were technical/IT problems, slightly more so for HCC (PC 44.5%; SC 40.1%) than for HC3 (PC 35.5%; SC 36.5%), disorganized information (PC 24.2%; SC 26.7% for HCC; PC 25.0%; SC 26.6% for HC3) and outdated information, slightly more so for HC3 (PC 18.8%; SC 18.4%) than for HCC (PC 13.8%; SC 15.5%), followed by insufficient knowledge of the program (PC 5.4%; SC 8.1% for HC3 and PC 5.8%; SC 9.2% for HCC) (Figure 1a,b).

A considerable proportion of doctors, higher in PC than SC, reported difficulties in cross-level consultations, more so for those conducted online (through EMR, PC 71.55%; SC 63.93%, and via email, PC 60.18%; SC 45.80%) than via phone (PC 62.76%; SC 44.29%) (Table 2). The type of difficulty also differed. While over all the answers, PC doctors most frequently reported late responses from SC doctors (36% through EMR and 37.0% via mail) and their failure to respond (37.6% via mail and 80.8% via phone), SC doctors reported PC doctors’ failure to provide sufficient relevant information to give an adequate reply (49.5% through EMR and 37.5% via mail) and their lack of response (59% for phone) (Figure 1c–e).

Most doctors of both care levels (78.71%) reported difficulties in participating in joint clinical case conferences (Table 2), but differed in the most frequently reported type of difficulty. While PC doctors reported limited or poor participation (34.1%), SC doctors reported timetable incompatibility (28.5%) and difficulties in arriving on time for the session (29%) (Figure 1f). 

### 3.4. Suggestions for Improvement of Cross-Level Clinical Coordination

**Figure 2:**
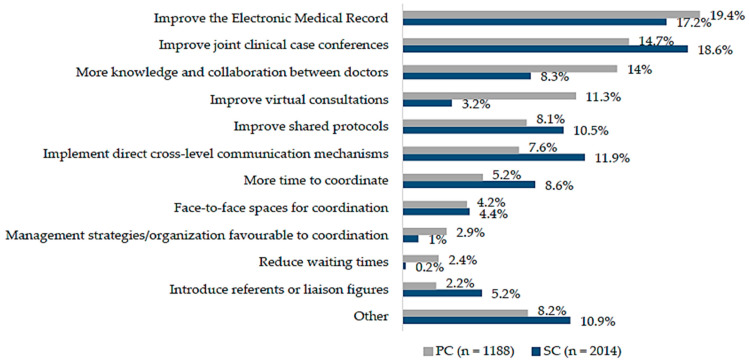
Suggestions for improvement of clinical coordination, by level of care; *n*: total of answers.

The suggestions for improving clinical coordination across levels were quite similar between PC and SC doctors, with slightly different frequencies, and were related to the extension, introduction or enhancement of different types of coordination mechanisms, mainly the EMR and joint clinical case conferences. Over the totality of answers, doctors referred to the need to improve the functioning of the shared EMR (PC 19.4%; SC 17.2%), by unifying systems, completing the information of the patient or better structuring and classifying the available information, and to improve joint clinical case conferences (PC 14.7%; SC 18.6%) by making them virtual, extending them to more specialities and adapting schedules. Suggestions also included the improvement of other coordination mechanisms, such as virtual consultations (PC 11.3%; SC 3.2%), by adding a notification system, and shared protocols (PC 8.1%; SC 10.5%), by unifying criteria or developing joint care circuits. Doctors also highlighted the implementation of direct cross-level communication mechanisms (PC 7.6%; SC 11.9%), such as the use of direct-messaging systems in clinical practice. In addition, doctors suggested fostering knowledge and collaboration between different care levels (PC 14%; SC 8.3%) by encouraging direct contact and teamwork. Other suggestions were related to organizational factors, such as having more time to coordinate (PC 5.2%; SC 8.6%) or introducing management strategies that favour coordination (PC 2.9%; SC 1%), as well as setting cross-level budgets and objectives (Figure 2). 

## 4. Discussion

This is the first study to conduct a comprehensive analysis of the use of a combination of clinical coordination mechanisms in a national health system. It fills a gap in the body of literature on the factors and difficulties preventing their appropriate use and also explores suggestions for improving clinical coordination, from the point of view of PC and SC doctors, so it may be relevant to policy makers around the globe in their attempts to improve clinical coordination for the benefit of patients, particularly in healthcare settings with similar characteristics.

### 4.1. Individual and Organizational Factors and Difficulties Underlying the (Low) Utilization of Coordination Mechanisms

The results obtained show that the level of use varies according to the mechanism and level of care. In keeping with the literature [36,37,38], the most frequently used mechanism is the EMR, especially among PC doctors. However, the results for the suggestions indicate that just sharing information through EMRs is not enough for the coordination of clinical care, especially for complex or urgent cases that require direct or rapid communication and feedback [39]. Doctors refer to the need to also implement direct communication mechanisms, which are precisely the type of mechanisms least used.

A major conclusion to draw from this study is that, as in other contexts [21,37], there is considerable room for improvement in the level of use of coordination mechanisms, especially in the use of the shared EMR of Catalonia (HC3) and joint clinical case conferences, mainly by SC doctors, and of the different cross-level consultations by both care levels. The lower use of HC3 could be explained by the greater number of difficulties identified by its users. However, since HC3 is the EMR shared by all providers in Catalonia, regardless of the ownership and management model, its implementation should be improved to enhance the coordination of information throughout the national health system. 

The analysis of both the factors influencing frequent use of coordination mechanisms and the reported difficulties in use give us some clues to understanding the organizational or individual reasons behind their low level of use, and the differences in use between levels of care. These elements need to be taken into account in the design of strategies aiming to improve the implementation and adoption of coordination mechanisms. 

Some factors to which particular attention should be devoted are those that are negatively associated with the use of most mechanisms for both levels of care, such as the type of healthcare area. Firstly, the negative association of the semi-integrated management model seems to be related to an unequal distribution of coordination efforts (common objectives, resources and coordination mechanisms between levels) among the PC centres of the territory, which focuses on the PC centres managed by the same entity as the hospital and excludes the rest [40,41]. Secondly, in areas with high technology hospitals, the greater size and technological level of the hospital may hinder coordination with the PC centres of its own area (in terms of time and available resources) [42], since these hospitals also cover complex cases from other areas. 

The factors associated with the level of use of coordination mechanisms by SC doctors are interactional, organizational and employment-related. Multicomponent strategies are therefore required to improve coordination at this level. In terms of interactional factors, knowing the PC doctors personally (for all the studied mechanisms), and perceiving that their daily practice influences the practice of the other level (for EMR and phone consultation), contribute to a more frequent use of the mechanism, as observed in other studies [25]. These factors probably generate a greater interest in coordinating the follow-up of patients with primary care. Organizational factors include performing patient consultations in PC centres, which brings doctors of different levels together and promotes collaboration [43] (especially fostering participation in joint clinical case conferences), and feeling that the organization sets objectives aimed at coordination (meaning common objectives, incentives, and mechanisms across levels [44]), for the different types of consultations. Finally, the lower use of mechanisms among SC doctors is associated with having more years of work experience (especially in the case of technology-based mechanisms, possibly related to the generational gap [45]), and with working in a surgical speciality (probably related to not knowing the PC doctors personally or less need to coordinate with each other) [46]. 

In contrast, PC doctors’ use of mechanisms is less clearly associated with individual factors (interactional or work-related), with the exception of their self-recognition as patient care coordinators for asynchronous consultations, and more with organizational factors (specifically, with the type of area). Although further research is required, one possible explanation is that the need for coordination is higher among PC doctors since their care practice requires continuous coordination of information and patient management with many specialists at the same time. Therefore, their use of coordination mechanisms does not depend so much on individual interest, but on organizational will to make them available.

Furthermore, the unexpected negative association of the opinion that managers facilitate coordination with the frequent use of most of the mechanisms among both care levels could be related to the difficulties experienced in the use of coordination mechanisms by doctors who use them more. The difficulties in use of the coordination mechanisms differed according to each mechanism. In the case of EMRs (HC3 and HCC), the main difficulties identified were technical problems and disorganized and outdated information, which have also been identified in other studies [28,47], and associated with a lack of managerial support [48]. Moreover, in line with findings in other studies [49,50], users of the different consultation types experienced great difficulties in contacting the doctor of the other level in a timely manner, and this may be due to the lack of an effective notification system and lack of time to reply, which again are issues that can be solved with institutional support. Another reported difficulty in use of consultations is not being provided with enough relevant information to give an adequate response, which should be attributed to a lack of professional will [50]. Furthermore, frequent participation in joint clinical case conferences is hindered by considerable difficulties related to incompatible schedules and the travel time required, in keeping with the literature [27]. One possible solution, beyond institutional support for timetable compatibility [51], might be to convert them to a virtual format [52].

### 4.2. Three Routes Targeting Clinical Coordination: Improve Coordination Mechanisms, More Interaction between Care Levels and Institutional Support

Doctors from PC and SC are the main actors in cross-level clinical coordination, thus their perspectives are crucial in order to better understand and improve this phenomenon. The suggestions they made in this study for improving clinical coordination were similar for both care levels and consistent with the difficulties and factors associated with the use of coordination mechanisms. They can be classified into three types: related to coordination mechanisms (improve existing mechanisms, and implement new direct communication mechanisms such as instant messaging applications); related to interactional factors (encourage personal knowledge and collaboration, and create face-to-face spaces for coordination); and related to organizational factors (more time to coordinate, management strategies that favour coordination, and reduction of waiting times). These results are consistent with previous studies that identify these factors as effective for improving the experience and perception of clinical coordination [13,14,33]. The fact that PC and SC doctors make similar suggestions for improving clinical coordination indicates their feasibility [53]. By following these three suggested routes, which contain elements that are rarely considered in organizational strategies, such as enhancing direct communication and mutual knowledge between care levels, managerial support could have a significant impact on improving clinical coordination. 

### 4.3. Limitations

The main limitation of this study is potential selection bias due to the self-administered nature of the questionnaire. However, the data on characteristics such as sex, age, and level of care of the universe of doctors of the Catalan national health system are similar to those of the study sample (Table S1) [54].

## 5. Conclusions

In the current era where health care is moving into the digital world, the managerial will to improve clinical coordination relies increasingly on the adoption of health information technologies. However, the evaluation of clinical coordination mechanisms –including those based on new technologies– through a comprehensive approach is limited. This study contributes to identifying the difficulties and factors affecting the use of several cross-level clinical coordination mechanisms, and it may therefore be useful at both a local and worldwide level for policy makers attempting to implement strategies to improve their use and effectiveness. To fulfil this goal, in accordance with doctors’ suggestions, particular attention should be paid to organizational factors such as managerial support to create the appropriate conditions for their use (in terms of mechanism content, infrastructure and availability of time). It is important, moreover, to strengthen interaction between physicians to improve the use of coordination mechanisms, especially among SC doctors, who are the most reluctant to use them, and to design specific strategies to promote their use among doctors with more work experience or working in a surgical speciality. Finally, the results also indicate that in order to improve clinical coordination in national health systems, special efforts should be made to implement coordination mechanisms uniformly across all the providers of a given healthcare area, and especially in areas where the reference hospital is a high technology hospital.

## Figures and Tables

**Table 1:** Description of the sample characteristics, by level of care.

	Total (*n* = 3308)	Primary Care (*n* = 1141)	Secondary Care (*n* = 2167)	
	*n* (%)	*n* (%)	*n* (%)	*p*
Sociodemographic characteristics				
Sex				<0.001
Male	1214 (42.12)	307 (31.01)	907 (47.94)	
Female	1668 (57.88)	683 (68.99)	985 (52.06)	
Age				<0.001
25–40 years	701 (25.46)	180 (18.97)	521 (28.88)	
41–55 years	1278 (46.42)	486 (51.21)	792 (43.90)	
56–70 years	774 (28.11)	283 (29.82)	491 (27.22)	
Country of birth				0.345
Spain	2469 (87.37)	858 (88.18)	1611 (86.94)	
Other	357 (12.63)	115 (11.82)	242 (13.06)	
Medical speciality				<0.001
Clinical speciality	2165 (78.36)	956 (98.86) ^a^	1209 (67.32)	
Surgical speciality	264 (9.55)	0 (0)	264 (14.70)	
Medical and surgical speciality	334 (12.09)	11 (1.14)	323 (17.94)	
Employment characteristics				
Years working as a doctor				<0.001
0–10 years	378 (13.54)	88 (9.11)	290 (15.89)	
11–20 years	929 (33.29)	329 (34.06)	600 (32.88)	
21–30 years	805 (28.84)	318 (32.92)	487 (26.68)	
31–45 years	679 (24.33)	231 (23.91)	448 (24.55)	
Type of contract (a)				<0.001
Permanent	2630 (90.94)	965 (96.31)	1665 (88.10)	
Temporary	262 (9.06)	37 (3.69)	225 (11.90)	
Type of contract (b)				0.233
Full-time	2660 (91.88)	929 (92.71)	1731 (91.44)	
Part-time	235 (8.12)	73 (7.29)	162 (8.56)	
Attitude towards work				
Satisfaction with the job in the organization ^b^	2193 (84.80)	741 (82.24)	1452 (86.17)	0.008
Type of area				
Area according to type of management of PC and SC				0.001
Integrated	1457 (44.04)	477 (41.81)	980 (45.22)	
Semi-integrated	892 (26.96)	354 (31.03)	538 (24.83)	
Non-integrated	959 (28.99)	310 (27.17)	649 (29.95)	
Area according to type of hospital				<0.001
Local or regional hospitals	1857 (56.14)	770 (67.48)	1087 (50.16)	
High resolution regional hospitals	810 (24.49)	222 (19.46)	588 (27.13)	
High technology hospitals	641 (19.38)	149 (13.06)	492 (22.70)	
Interactional factors between doctors				
My experience of coordination with the other care level is positive ^c^	1864 (71.01)	701 (70.81)	1163 (71.13)	0.860
I know the doctors of the other care level who see my patients personally ^c^	1103 (39.06)	442 (44.02)	661 (36.32)	<0.001
I trust in the clinical skills of the doctors of the other level who see my patients ^c^	2429 (88.62)	971 (97.20)	1458 (83.70)	<0.001
My daily practice influences the practice of the doctors of the other level ^c^	1834 (78.61)	554 (66.19)	1280 (85.56)	<0.001
In practice, primary care doctors are responsible for coordinating the patient on their way through the different levels of care ^c^	2189 (81.92)	950 (95.19)	1239 (74.01)	<0.001
Organizational factors				
My organization’s management facilitates coordination between primary and secondary care doctors ^c^	1492 (59.18)	665 (67.44)	827 (53.88)	<0.001
My organization sets objectives that are aimed at coordination between care levels ^c^	1370 (55.20)	540 (56.72)	830 (54.25)	0.228
The time I can dedicate to coordinating with doctors of the other level during my working day is sufficient ^c^	380 (13.90)	136 (13.67)	244 (14.03)	0.792
As a secondary care doctor, do you do patient consultations in a primary care centre? ^c^			386 (21.44)	

^a^ Family doctors; ^b^ Results correspond to the category “yes”; ^c^ Results correspond to the categories “always” and “very often”.

**Table 2:** Frequency of use and existence of difficulties in use of clinical coordination mechanisms, by level of care.

	Total (*n* = 3308)	Primary Care (*n* = 1141)	Secondary Care (*n* = 2167)	
	*n* (%)	*n* (%)	*n* (%)	*p*
Shared EMR of Catalonia (HC3)				
Frequent use ^1^	1939 (65.89)	802 (77.34)	1137 (59.65)	<0.001
Existence of difficulties ^a^	2099 (76.89)	686 (74.89)	1413 (77.89)	0.079
Shared EMR of the organization (HCC)				
Frequent use ^1^	2044 (81.27)	836 (94.04)	1208 (74.29)	<0.001
Existence of difficulties ^a^	1774 (66.05)	590 (64.84)	1184 (66.67)	0.343
Virtual consultation through EMR				
Frequent use ^2^	854 (52.36)	430 (53.22)	424 (51.52)	0.492
Existence of difficulties ^a^	988 (67.67)	513 (71.55)	475 (63.93)	0.002
Email consultation				
Frequent use ^2^	460 (26.11)	179 (23.31)	281 (28.27)	0.019
Existence of difficulties ^a^	775 (52.12)	393 (60.18)	382 (45.80)	<0.001
Phone consultation				
Frequent use ^2^	425 (21.73)	86 (12.80)	339 (26.40)	<0.001
Existence of difficulties ^a^	755 (50.27)	305 (62.76)	450 (44.29)	<0.001
Joint clinical case conferences				
Frequent participation ^3^	748 (63.28)	473 (69.87)	275 (54.46)	<0.001
Existence of difficulties ^a^	865 (78.71)	498 (78.67)	367 (78.76)	0.974

Frequent use considered as ^1^ *“*Daily”, ^2^ “Daily or weekly” and ^3^ “Daily, weekly or monthly” (over the total who reported having access); ^a^ Results correspond to the category “Yes” (over the total of doctors).

**Table 3:** Factors associated with frequent use of the shared Electronic Medical Records (EMRs), by level of care.

	HC3 ^1^		HCC ^1^	
	Primary Care	Secondary Care	Primary Care	Secondary Care
	Adj OR (95% CI)	Adj OR (95% CI)	Adj OR (95% CI)	Adj OR (95% CI)
Sociodemographic characteristics				
Sex				
Male	1.22 (0.90–1.64)	0.92 (0.62–1.38)	0.71 (0.49–1.02)	0.82 (0.67–1.00)
Female	1	1	1	1
Country of birth				
Spain	0.63 (0.21–1.87)	0.66 (0.57–0.78)	1.36 (0.52–3.51)	1.00 (0.66–1.52)
Other	1	1	1	1
Medical speciality				
Clinical speciality		1		1
Surgical speciality		0.44 (0.24–0.80)		0.52 (0.34–0.79)
Medical and surgical speciality		0.47 (0.24–0.91)		0.66 (0.58–0.76)
Employment characteristics				
Years working as a doctor				
0 to 10 years	1	1	1	1
11 to 20 years	2.10 (1.55–2.84)	0.67 (0.48–0.94)	2.51 (1.31–4.79)	0.69 (0.50–0.96)
21 to 30 years	2.30 (2.08–2.54)	0.69 (0.52–0.93)	1.35 (0.80–2.30)	0.63 (0.45–0.89)
31 to 45 years	1.50 (1.38–1.63)	0.44 (0.34–0.58)	1.63 (0.57–4.62)	0.32 (0.17–0.60)
Type of contract (b)				
Part-time		1		
Full-time		1.58 (1.18–2.13)		
Satisfaction with the job in the organization				
Yes	0.46 (0.30–0.71)			
No	1			
Type of area				
Area according to type of hospital				
Local or regional hospitals	1	1	1	1
High resolution regional hospitals	0.66 (0.34–1.29)	0.81 (0.54–1.20)	1.01 (0.40–2.58)	1.08 (0.87–1.35)
High technology hospitals	0.88 (0.68–1.13)	0.67 (0.61–0.75)	0.98 (0.73–1.31)	0.53 (0.44–0.65)
Area according to type of management of PC and SC				
Integrated	1	1	1	1
Semi-integrated	1.69 (1.51–1.88)	1.48 (1.39–1.59)	0.80 (0.67–0.94)	0.70 (0.65–0.74)
Non-integrated	3.87 (3.30–4.54)	2.07 (1.93–2.22)	1.86 (1.73–2.00)	0.70 (0.65–0.74)
Interactional factors between doctors				
My experience of coordination with the other care level is positive				
Rarely/Never	1			
Often/Always	0.59 (0.46–0.75)			
I know the doctors of the other care level who see my patients personally				
Rarely/Never		1	1	1
Often/Always		1.16 (1.07–1.26)	0.60 (0.51–0.71)	1.43 (1.11–1.84)
My daily practice influences the practice of the doctors of the other level				
Rarely/Never	1			1
Often/Always	0.52 (0.47–0.58)			1.39 (1.18–1.64)
Organizational factors				
My organization’s management facilitates cross-level coordination				
Rarely/Never		1	1	
Often/Always		0.80 (0.68–0.94)	0.58 (0.42–0.80)	
The time I can dedicate to coordinating with other level doctors during my working day is sufficient				
Rarely/Never	1			
Often/Always	0.63 (0.52–0.76)			

^1^ Frequent use considered “Daily”.

**Table 4:** Factors associated with the frequent use of virtual consultation through EMR, email and phone consultation, by level of care.

	Virtual Consultation through EMR ^1^		Email Consultation ^1^		Phone Consultation ^1^	
	Primary Care	Secondary Care	Primary Care	Secondary Care	Primary Care	Secondary Care
	Adj OR (95% CI)	Adj OR (95% CI)	Adj OR (95% CI)	Adj OR (95% CI)	Adj OR (95% CI)	Adj OR (95% CI)
Sociodemographic characteristics						
Sex						
Male	0.98 (0.76–1.26)	1.73 (1.55–1.92)	1.11 (0.65–1.90)	1.58 (1.47–1.69)	0.60 (0.35–1.01)	1.29 (1.20–1.38)
Female	1	1	1	1	1	1
Country of birth						
Spain	1.29 (0.72–2.32)	1.73 (1.29–2.32)	0.66 (0.41–1.06)	1.25 (0.74–2.12)	0.40 (0.32–0.50)	1.10 (0.73–1.66)
Other	1	1	1	1	1	1
Medical speciality						
Clinical speciality		1		1		1
Surgical speciality		0.61 (0.45–0.82)		0.30 (0.15–0.59)		0.20 (0.10–0.39)
Medical and surgical speciality		1.21 (0.76–1.93)		0.74 (0.64–0.86)		0.65 (0.40–1.05)
Employment characteristics						
Years working as a doctor						
0 to 10 years	1	1	1	1	1	1
11 to 20 years	0.97 (0.45–2.10)	0.65 (0.36–1.17)	0.76 (0.68–0.84)	0.69 (0.38–1.27)	1.30 (0.46–3.69)	0.92 (0.72–1.16)
21 to 30 years	0.91 (0.35–2.36)	0.56 (0.46–0.68)	0.65 (0.50–0.85)	0.57 (0.47–0.69)	0.80 (0.17–3.74)	0.99 (0.80–1.21)
31 to 45 years	0.54 (0.29–1.01)	0.44 (0.43–0.45)	0.63 (0.36–1.09)	0.63 (0.51–0.79)	0.87 (0.21–3.58)	0.97 (0.86–1.08)
Type of contract a)						
Permanent						1
Temporary						2.15 (1.12–4.14)
Type of area						
Area according to type of hospital						
Local or regional hospitals	1	1	1	1	1	1
High resolution regional hospitals	1.41 (0.42–4.77)	1.72 (1.41–2.08)	0.70 (0.41–1.18)	0.69 (0.18–2.62)	1.45 (0.80–2.61)	0.68 (0.27–1.71)
High technology hospitals	0.63 (0.40–0.99)	0.95 (0.81–1.12)	0.44 (0.37–0.52)	0.57 (0.34–0.95)	0.58 (0.44–0.76)	0.55 (0.33–0.93)
Area according to type of management						
Integrated	1	1	1	1	1	1
Semi-integrated	0.68 (0.53–0.86)	1.23 (1.13–1.35)	0.59 (0.52–0.67)	0.58 (0.45–0.72)	1.51 (1.39–1.64)	1.12 (0.90–1.39)
Non-integrated	0.77 (0.60–1.00)	1.31 (1.27–1.35)	0.76 (0.67–0.87)	0.93 (0.85–1.02)	1.30 (1.16–1.45)	0.76 (0.70–0.83)
Interactional factors between doctors						
I know the doctors of the other care level who see my patients personally						
Rarely/Never		1		1		1
Often/Always		1.47 (1.08–2.00)		2.05 (1.61–2.60)		1.98 (1.27–3.09)
I trust in the clinical skills of the doctors of the other level who see my patients						
Rarely/Never			1			
Often/Always			1.84 (1.19–2.85)			
My daily practice influences the practice of the doctors of the other level						
Rarely/Never			1			1
Often/Always			1.33 (1.10–1.62)			2.86 (1.55–5.28)
PC doctors are responsible for coordinating the patient on their way through the different levels of care						
Rarely/Never	1		1			
Often/Always	1.31 (1.08–1.60)		1.83 (1.47–2.28)			
Organizational factors						
My organization’s management facilitates coordination between primary and secondary care doctors						
Rarely/Never				1		1
Often/Always				0.65 (0.43–0.98)		0.60 (0.56–0.64)
My organization sets objectives aimed at coordination between care levels						
Rarely/Never		1		1		1
Often/Always		1.65 (1.41–1.94)		1.53 (1.14–2.06)		2.02 (1.46–2.79)
Patient consultations in a primary care centre by SC doctors						
Rarely/Never		1				
Often/Always		1.57 (1.13–2.17)				

^1^ Frequent use considered “Daily or weekly”.

**Table 5:** Factors associated with frequent participation in joint clinical case conferences, by level of care.

	Joint Clinical Case Conferences ^1^	
	Primary Care	Secondary Care
	Adj OR (95% CI)	Adj OR (95% CI)
Sociodemographic characteristics		
Sex		
Male	0.86 (0.51–1.43)	0.64 (0.51–0.79)
Female	1	1
Country of birth		
Spain	1.00 (0.66–1.47)	0.40 (0.24–0.66)
Other	1	1
Employment characteristics		
Years working as a doctor		
0–10 years	1	1
11–20 years	0.75 (0.22–2.57)	0.87 (0.54–1.39)
21–30 years	0.70 (0.18–2.68)	0.66 (0.39–1.11)
31–45 years	0.58 (0.23–1.47)	0.86 (0.48–1.55)
Type of contract b)		
Part-time		1
Full-time		1.98 (1.06–3.71)
Type of area		
Area according to type of hospital		
Local or regional hospitals	1	1
High resolution regional hospitals	1.37 (0.69–2.70)	1.38 (0.77–2.48)
High technology hospitals	1.02 (0.94–1.10)	0.74 (0.58–0.95)
Area according to type of management of PC and SC		
Integrated	1	1
Semi-integrated	0.99 (0.93–1.05)	0.82 (0.79–0.85)
Non-integrated	1.19 (1.10–1.28)	1.50 (1.39–1.61)
Interactional factors between doctors		
My experience of coordination with the other care level is positive		
Rarely/Never	1	
Often/Always	1.82 (1.51–2.21)	
I know the doctors of the other care level who see my patients personally		
Rarely/Never		1
Often/Always		2.79 (1.70–4.61)
I trust in the clinical skills of the doctors of the other level who see my patients		
Rarely/Never	1	
Often/Always	0.37 (0.14–0.96)	
Organizational factors		
Patient consultations in a primary care centre by SC doctors		
Rarely/Never		1
Often/Always		1.33 (1.07–1.64)

^1^ Frequent use considered “Daily, weekly or monthly”.

## Data Availability

The datasets generated and/or analyzed in this study are not publicly available because individual privacy could be compromised, but they are available from the corresponding author on reasonable request.

## Supplementary Materials

The following are available online at https://www.mdpi.com/1660-4601/18/6/3224/s1, Table S1: Distribution of some characteristics of the doctors of the Catalan national health system (NHS).
