# Supplementary material for: Understanding How to Improve the Use of Clinical Coordination Mechanisms between Primary and Secondary Care Doctors: Clues from Catalonia
Source: Int J Environ Res Public Health. 2021 Mar 20;18(6):3224. doi: 10.3390/ijerph18063224 (PMC8003988; doi:10.3390/ijerph18063224)
Supplement: Supplementary file 1 [file ijerph-18-03224-s001.pdf]

International Journal of
Environmental Research and Public Health

MDPI

Article

Understanding How to Improve the Use of Clinical Coordination Mechanisms between Primary and Secondary Care Doctors: Clues from Catalonia

Laura Esteve-Matalí [1,2], Ingrid Vargas [1,*], Franco Amigo [1,3], Pere Plaja [4], Francesc Cots [5], Erick F. Mayer [6], Joan-Manuel Pérez-Castejón [7] and María-Luisa Vázquez [1]

1 Health Policy and Health Services Research Group, Health Policy Research Unit, Consortium for Health Care and Social Services of Catalonia, 08022 Barcelona, Spain; lesteve@consorci.org (L.E.-M.); famigo@imim.es (F.A.); mlvazquez@consorci.org (M.-L.V.)
2 Department for Paediatrics, Obstetrics and Gynaecology, Preventive Medicine, Universitat Autònoma de Barcelona, 08193 Bellaterra, Spain
3 Health Services Research Unit, IMIM-Institut Hospital del Mar d'Investigacions Mèdiques, 08003 Barcelona, Spain
4 Fundació Salut Empordà, 17600 Figueres, Spain; pplaja@salutemporda.cat
5 Parc de Salut Mar, 08019 Barcelona, Spain; FCots@parcdesalutmar.cat
6 Serveis de Salut Integrats Baix Empordà, 17230 Palamós, Spain; emayer@ssibe.cat
7 Badalona Serveis Assistencials, 08911 Badalona, Spain; jpcastejon@bsa.cat
* Correspondence: ivargas@consorci.org

Table S1. Distribution of some characteristics of the doctors of the Catalan national health system (NHS).

	Doctors of the Catalan NHS
**Sex**	
Male	55.1%
Female	44.9%
**Age** (years old)	
Mean	44.6
**Level of care**	
PC	27.4%
SC	72.6%

Source: Solsona et al. [54].
